# Supplementary figures and images for: A Minimal Model of Signaling Network Elucidates Cell-to-Cell Stochastic Variability in Apoptosis
Source: PLoS One. 2010 Aug 11;5(8):e11930. doi: 10.1371/journal.pone.0011930 (PMC2920308; doi:10.1371/journal.pone.0011930)

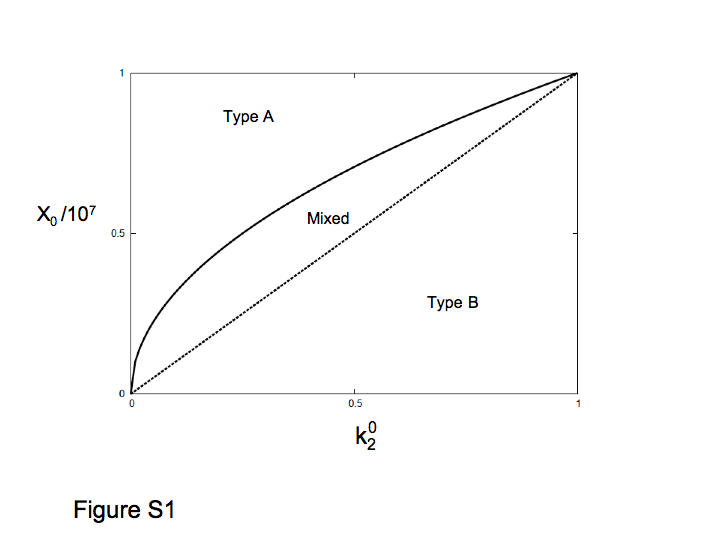

Supplement: Figure S1 — Differential signaling response of the minimal network as k2 0 and x0 are varied. This phase diagram is generated using two relations obtained from a parameter sensitivity analysis of the minimal model: (i) type A signaling dominates for all times when x0>k32 0 √k2 0x1 0x2 0/k31 0, (ii) type B signaling dominates from the beginning when x0<k2 0x1 0x2 0/k31 0x3 0. Parameter values used here are x1 0 = 100, x2 0 = 100, x3 0 = 100, k31 0 = 10−5, and k32 0 = 1.0. (0.04 MB TIF) [file pone.0011930.s001.tif]
